# The Role of Contactin 1 in Cancers: What We Know So Far

**DOI:** 10.3389/fonc.2020.574208

**Published:** 2020-10-29

**Authors:** Yumei Liang, Cui Ma, Fengjuan Li, Guanhua Nie, Haining Zhang

**Affiliations:** ^1^ Department of Neurology and Neuroscience Center, The First Hospital of Jilin University, Changchun, China; ^2^ Department of Pediatric Hematology, The First Hospital of Jilin University, Changchun, China; ^3^ Oncology Department of Tumor Center, The First Hospital of Jilin University, Changchun, China

**Keywords:** contactin 1 (CNTN1), cancers, epithelial-mesenchymal transition (EMT), PI3K/AKT, signal transduction pathways, targeted therapy

## Abstract

Cancers are among the difficult-to-treat diseases despite advances in diagnosis and treatment. Although newer effective targets remain to be discovered, targeted therapy has emerged as a promising field. In the last decade, contactin 1 (CNTN1) has surfaced as an important cancer-related molecule. CNTN1 is a neuronal membrane glycoprotein, which, if overexpressed, is found in different cancer cell lines, cancer tissues, and transgenic mice. It is positively associated with lymphatic invasion, metastasis, late TNM stage, and a short overall survival time. However, the role of CNTN1 in cancer cell proliferation remains unclear. In addition, CNTN1 is involved in cancer cell invasion, migration, metastasis, and chemoresistance by promoting epithelial-mesenchymal transition and mediating several signal transduction pathways. Several studies suggest CNTN1 as a new therapeutic target for cancers. This review aims to summarize the research developments on CNTN1 in various cancers, to establish its role in epithelial-mesenchymal transition and signal transduction pathways, and to identify promising areas for further investigation.

## Introduction

Cancer remains a persistent health problem in society, and targeted therapy is an effective measure for treatment. Contactin 1 (CNTN1) is a neuronal membrane glycoprotein belonging to the cell adhesion molecules and immunoglobulin superfamily. CNTN1 has been demonstrated as an essential component in oligodendrocyte differentiation and maturation (1, 2), neurogenesis (3), axon guidance and formation (4), neurotransmitter release (5), and synaptic plasticity (6, 7). The *CNTN1* gene is mapped to human chromosome 12q11-q12, which is close to a breakpoint region of cancers (8). After Su et al. initially found CNTN1 to be associated with lung cancer (9), the relationship between CNTN1 and cancer has been studied in different cancer types, along with related molecules and signal transduction pathways. CNTN1 expression is upregulated at the transcriptional and translational levels in various cancer cell lines, cancer tissues, and transgenic mice. It is associated with invasion, migration, metastasis, and poor prognosis in several cancers originating in organs, such as the lung, stomach, prostate, esophagus, oral cavity (oral squamous cell carcinoma, OSCC), thyroid, liver, and breast. Moreover, CNTN1 promotes cell invasion, migration, and metastasis *via* the epithelial-mesenchymal transition (EMT) in gastric, lung, and prostate cancers and is defined as an independent prognostic predictor of gastric cancer (10–12). CNTN1 also contributes to chemoresistance in lung cancer (13–15). Additionally, CNTN1 is associated with several signal transduction pathways, such as the phosphatidylinositol 3-kinase (PI3K)/AKT, vascular endothelial growth factor-C (VEGF-C)/vascular endothelial growth factor receptor-3 (VEGFR-3), α7 nicotinic acetylcholine receptor (nAChR)/extracellular signal-regulated kinase (ERK), member A of the Ras homolog gene family (RhoA), Src-p38 mitogen-activated protein kinase (MAPK)-CCAAT/enhancer-binding protein (C/EBP) α, Notch1, and Ret protooncogene and Ret-activating protein ELE1 (RET/PTC3). Thus, CNTN1 is a promising candidate for cancer-targeted therapy (12–21).

## Expression of CNTN1 in Cancer Tissues and Its Correlation With Clinicopathological Features

CNTN1 expression was significantly higher in different cancer tissues than in noncancerous controls. An immunohistochemical analysis of 105 specimens showed that CNTN1 expression was 2.65 times more positive in primary gastric cancer patients than in the noncancerous control group, and it was almost exclusively observed in the cytoplasm of cancer cells (22). Similarly, the expression of CNTN1 protein was found in 81% and 54.4% of 100 thyroid cancer tissues and 90 hepatocellular carcinoma (HCC) samples, respectively. These were compared with the corresponding paracancerous tissues with 10% and 12.2% (19, 20). CNTN1 expression was more easily detected in prostate cancer cells, in both primary tumors and metastases, than in normal prostate glands or paracancerous tissues (16, 23). The CNTN1 positivity rate increased as the corresponding tissues progressed from nontumor prostate tissues to high-grade prostatic intraepithelial neoplasia and to carcinomas (16, 23). Compared to 30 normal esophageal tissue samples, 82 primary esophageal cancer tissue samples had a significantly increased expression of CNTN1 mRNA and an overall stronger immunohistochemical staining for CNTN1 protein (18). In the immunohistochemical analysis of 55 surgical specimens from patients with tongue squamous cell carcinoma, CNTN1 expression was 52.7% positive and was positively associated with neck metastasis, vascular invasion, and lymphatic invasion (17).

Clinical and experimental studies positively correlated the overexpression of *CNTN1* with cancer progression and a poor prognosis. In the immunohistochemical analysis of specimens from gastric cancer, lung adenocarcinoma, primary OSCC, prostate cancer, and esophageal cancer, CNTN1 expression was significantly positively associated with lymphatic invasion, metastasis, late TNM stage, and a short overall survival time (9, 12, 13, 16, 18, 22–25). However, it was not associated with vascular or serosa invasion. Additionally, CNTN1 was positively correlated with the late TNM stage in thyroid cancer tissues (19). In HCC tissues, it was correlated with the late TNM stage and a short overall and disease-free survival time (20). In transgenic mice injected with prostate cancer cells overexpressing CNTN1, xenograft tumorigenesis, lung metastases, and metastatic nodules were increased (23). In transgenic mice injected with gastric cancer or lung adenocarcinoma cells where *CNTN1* was knocked down, the number of lung metastases and metastatic nodules was significantly smaller than that of the control (9, 12). This suggests that CNTN1 is essential for tumor metastasis.

CNTN1 was consistently involved in cancer progression across different cancer types, but its role in cancer cell proliferation is contradictory. In lung adenocarcinoma, gastric cancer, and OSCC, knockdown of *CNTN1* strongly impaired cell migratory and invasive capacities *in vitro* without influencing proliferation (12–14, 25, 26). In *in vivo* studies, the weight and volume of the tumors, obtained from transgenic mice injected with gastric cancer cells with a knocked down *CNTN1* expression were almost identical to those from the control group (12). This suggests that CNTN1 is essential for tumor metastasis, but not tumor formation and proliferation. However, *in vitro* knockdown of *CNTN1* inhibited cancer cell proliferation, colony formation, migration, and invasion, whereas overexpression of *CNTN1* increased tumor xenograft formation and metastasis in prostate cancer (16, 23). In contrast, ectopic expression of *CNTN1* in DU145 prostate cancer cells displayed no effect on cell proliferation but enhanced cell invasion (23). Additionally, expression of *CNTN1* was associated with cell proliferation in esophageal, thyroid, and breast cancers (19, 21, 27). The volume and weight of xenograft tumors from nude mice, transfected with breast cancer cells overexpressing CNTN1, were significantly higher than those from the control (21). This suggests that a higher expression of CNTN1 promotes proliferation and tumorigenicity. In summary, the role of CNTN1 in tumor proliferation varies across different tumors although it appears inconsistent in prostate cancer. Moreover, regulation of tumor proliferation requires a complex signaling network involving several molecules and pathways along with complementary and counteracting mechanisms in different cancer types. Thus, future studies should focus on identifying correlated molecular mechanisms to ascertain the association between CNTN1 expression and tumor proliferation.

## CNTN1 Facilitates Invasion, Migration, Metastasis, and Chemoresistance Through Promoting the EMT Process *via* Activating PI3K/AKT Pathway in Cancers

EMT refers to the phenotypic transformation from the epithelial to mesenchymal cells. This phenomenon is pivotal in tumor cell invasion, migration, metastasis, and drug resistance (28). EMT-related markers include epithelial markers, such as E-cadherin; mesenchymal markers, such as N-cadherin, vimentin, and fibronectin; and related transcription factors, such as Snail, Slug, ZEB, and Twist (29). *CNTN1* has been described as an EMT-related gene in the EMT gene database (10). In 63 primary lung cancer samples, Yan et al. observed that 65% of the tissues were CNTN1 positive. Among them, 61% were E-cadherin negative. In addition, a negative correlation between CNTN1 and E-cadherin was observed as E-cadherin could be detected in the CNTN1 negative regions although it was downregulated in the CNTN1 positive tissues (26). Further, in the drug-resistant cell lines derived from small cell lung cancer (SCLC) and lung adenocarcinoma, the CNTN1 and p-AKT levels were increased upon N-cadherin and vimentin upregulation and E-cadherin downregulation. Knockdown of *CNTN1* suppressed p-AKT, N-cadherin, and vimentin levels and increased E-cadherin levels (14, 15). The inhibition of the AKT pathway in drug-resistant lung cancer cells using LY294002 conferred the same result without influencing CNTN1 expression (14, 15). Moreover, after inhibiting AKT activation, the knockdown of *CNTN1* failed to further reduce the invasive ability of cancer cells (26). This confirmed that CNTN1 was upstream to the PI3K/AKT pathway and promoted EMT by activating this pathway. Furthermore, knockdown of *CNTN1* in prostate cancer cell lines also inhibited the PI3K/AKT pathway and suppressed the EMT process *via* concomitant upregulation of E−cadherin and downregulation of N-cadherin and vimentin (16, 23). Additionally, knockdown of *CNTN1* resulted in an increased expression of the PH domain and leucine-rich repeat protein phosphatase 2 (PHLPP2) but not phosphatase and tensin homolog (PTEN). This suggests that CNTN1 partially enhances the activation of AKT by inhibiting PHLPP2-dependent dephosphorylation (26).

Furthermore, knockdown of *CNTN1* in lung cancer cells decreased the expression of SIP1 and Slug but not Snail (26), which is the most studied E-cadherin transcription inhibitor (30). A comparative analysis between the mRNA and protein expression levels of CNTN1 and EMT-related proteins (E-cadherin, Snail, Slug, and N-cadherin) indicated that CNTN1 induced EMT by activating the transcription factor Slug but not Snail in gastric cancer cells. Meanwhile, the E-cadherin levels were high in less invasive gastric cancer cells. Conversely, it was low in highly invasive gastric cancer cells. Opposing levels of Slug and N-cadherin were observed. The expression of Snail was almost identical between the less and highly invasive gastric cancer cells. However, knockdown of *CNTN1* in cancer cells led to a significant reduction in CNTN1, Slug, and N-cadherin expression as well as E-cadherin upregulation. Nevertheless, expression of Snail remained unaffected (12). These studies suggest the facilitative role of CNTN1 in EMT through specific transcription factors and its heterogeneous role in tumor progression.

CNTN1 expression at the mRNA and the protein levels was significantly higher in cisplatin-resistant lung adenocarcinoma cells than in the control (13). Furthermore, knockdown of *CNTN1* attenuated cancer progression and significantly increased drug sensitivity and cisplatin-induced apoptosis in both cisplatin-resistant cancer cells and the control (13, 14). In *in vivo* studies, *CNTN1* knockdown in a xenograft mouse model of cisplatin-resistant lung adenocarcinoma also reversed cisplatin resistance and reduced the metastasis potency (14). CNTN1 was also reported to enhance BCT-100 resistance in SCLC *in vitro* (15). BCT-100 is a chemotherapeutic drug from pegylated recombinant human arginase 1. The underlying mechanism behind this finding was related to the EMT process, wherein the PI3K/AKT pathway was activated (14, 15). This suggests the role of CNTN1 as a potential biomarker to predict and evaluate the chemotherapeutic efficacy, especially in patients with lung adenocarcinoma.

## Other Signal Transduction Pathways Associated With CNTN1

Several pathways other than the PI3K/AKT are involved in tumor progression, and the correlations between CNTN1 and the molecules in these pathways are summarized in **Table 1**. The VEGF-C/VEGFR-3 pathway is essential for lymphangiogenesis and lymphatic metastasis in various cancers (34). It plays a critical role in proliferation, migration, and invasion in cancers (35). Further, CNTN1 also correlates with cancer invasion, migration, and metastasis, especially to the lymph nodes (12, 13, 17, 18, 22, 25). The association between CNTN1 and the VEGF-C/VEGFR-3 pathway was first confirmed by Su et al. in lung adenocarcinoma (9). Overexpression of VEGF-C significantly increased the expression of CNTN1 and phosphorylation of p38 MAPK, which could be reversed by inhibition of VEGFR-3 or knockdown of *MAPK14*. The p38 MAPK inhibitor SB203580 also decreased the expression of CNTN1, indicating that the VEGF-C/VEGFR-3 pathway induced the expression of CNTN1 *via* phosphorylation of p38 MAPK. Further, the Src kinase inhibitor PP1, but not the MEK inhibitor PD98059, caused a marked decrease in p38 MAPK activation and CNTN1 expression. Moreover, a point mutation in the C/EBP response element reduced 80% of the CNTN1 promoter activity induced by VEGF-C, which could be abolished by SB203580 or PP1. This suggests that the VEGF-C/VEGFR-3 pathway induces the expression of CNTN1 *via* the Src-p38 MAPK-C/EBPα pathway. Furthermore, the binding of C/EBP with the *CNTN1* promoter was regulated by VEGF-C in esophageal cancer *in vitro* (27). A positive correlation between the expression of hypoxia-inducible factor-1α (HIF-1α), VEGF-C, and CNTN1 was also established (18). In 30 paired gastric adenocarcinoma samples, Liu et al. found a significant correlation between the expression of the sine oculis homeobox homolog 1 (SIX1), VEGF-C, and CNTN1 (24). However, a thorough correlation analysis between the CNTN1 and VEGF-C/VEGFR-3 pathways and related mediators has not yet been performed.

**Table 1:** Correlations between CNTN1 and other molecules in different cancers.

Tumors	Authors/Year	Cancer cell lines	Correlations	Ref
Lung cancer	Xu et al./2019	H446 and H526	CNTN1→AKT→N-cadherin CNTN1→AKT→vimentin CNTN1→AKT→→E-cadherin	(15)
	Zhang et al./2017	A549	CNTN1→PI3K/AKT→N-cadherin CNTN1→PI3K/AKT→vimentin CNTN1→PI3K/AKT→→E-cadherin	(14)
	Yan et al./2013	A549	CNTN1→→PHLPP2→→AKT→→E-Cadherin CNTN1→Slug and/or SIP1→→E-Cadherin	(26)
	Hung et al./2009	CL1.0	NNK→α7 nAChR/ERK→CNTN1	(31)
	Su et al./2006	A549 and H928	VEGF-C/VEGFR-3→Src-p38 MAPK-C/EBPα→CNTN1	(9)
Gastric cancer	Yang et al./2015	MKN45	HIF-1α→CNTN1→p115 RhoGEF→RhoA	(32)
	Chen et al./2015	MKN45	CNTN1→Slug→→E-Cadherin CNTN1→Slug→N-cadherin	(12)
	Liu et al./2014	NU	*SIX1→VEGF-C→CNTN1	(24)
	Qin et al./2011	MKN45	VEGFR-3→CNTN1	(33)
Prostate cancer	Wang et al./2020	PC3 and LNCaP	CNTN1→PI3K/AKT CNTN1→N-cadherin CNTN1→vimentin CNTN1→→E-cadherin	(16)
	Yan et al./2016	DU145	CNTN1→PI3K/AKT CNTN1→→E-cadherin	(23)
Esophageal cancer	Liu et al./2012	NU	*HIF-1α→VEGF-C→CNTN1	(18)
	Liu et al./2011	TE-1	*VEGF-C/VEGFR-2 and/or VEGFR-3→C/EBP→CNTN1	(27)
OSCC	Shigetomi et al./2018	SAS and HO1U1	VEGF-C/VEGFR-3→CNTN1	(17)
Thyroid cancer	Shi et al./2015	B-CPAP and BHT101	RET/PTC3→CNTN1 CNTN1→Notch1→CCND1	(19)

CNTN1, contactin 1; OSCC, oral squamous cell carcinoma; PI3K, phosphatidylinositol 3-kinase; PHLPP2, PH domain and leucine-rich repeat protein phosphatase 2; NNK, 4-(methylnitrosamino)-1-(3-pyridyl)-1-butanone; α7 nAChR, α7 nicotinic acetylcholine receptor; ERK, extracellular signal-regulated kinase; VEGF-C, vascular endothelial growth factor-C; VEGFR-3, vascular endothelial growth factor receptor-3; p38 MAPK, p38 mitogen-activated protein kinase; C/EBP, CCAAT/enhancer-binding protein; HIF-1α, hypoxia-inducible factor-1α; p115 RhoGEF, p115 Rho guanine nucleotide exchange factor; RhoA, member A of the Ras homolog gene family; SIX1, sine oculis homeobox homolog 1; VEGFR-2, vascular endothelial growth factor receptor-2; RET/PTC3, Ret protooncogene and Ret-activating protein ELE1; CCND1, cyclin D1; Ref, references; NU means no cancer cell lines was used but cancer tissues in the study; Single arrow represents stimulatory effect, and two successive arrows represent inhibitory effect; The correlations with * were confirmed with insufficient evidence and reasonable speculation in the studies.

Next, Hung et al. found that 4-(methylnitrosamino)-1-(3-pyridyl)-1-butanone (NNK) induced CNTN1 *via* the α7 nAChR/ERK pathway in lung adenocarcinoma cells (31). NNK is a carcinogen found in tobacco. In lung cancer cells, NNK directly increased the expression of CNTN1, at both the transcriptional and translational levels and activated α7 nAChR downstream to the AKT and ERK pathways. Further, inhibition of both nAChR by α-bungarotoxin and the ERK signaling pathway by PD98059 could reverse the increased expression of CNTN1 induced by NNK. Inhibition of the AKT pathway with LY294002 had no effect on CNTN1 expression. This indicated that NNK promoted lung cancer progression by upregulating CNTN1 expression *via* the α7 nAChR/ERK pathway but not the α7 nAChR/AKT pathway. Additionally, the expression of nAChR was not affected by treatment with NNK in lung cancer cells, indicating that the effect of NNK was only *via* receptor binding. Moreover, treatment with NNK led to a sixfold increase in cell invasion and a 30% increase in cell adhesion. However, treatment with anti-CNTN1 antibodies inhibited cell invasion by 70%–80%, suggesting a critical role of CNTN1 in mediating cancer invasion induced by NNK.

Further, CNTN1 was found to be associated with the RhoA, Src-p38 MAPK-C/EBPα, Notch1, and RET/PTC3 pathways in cancers. The knockdown of *CNTN1* reduced cell migration and suppressed activation of RhoA and phosphorylation of p115 Rho guanine nucleotide exchange factor (RhoGEF, a RhoA activator) in gastric cancer. However, this effect could be reversed by activating RhoA. Consequently, RhoA was a downstream factor to CNTN1 (32). Moreover, knockdown of *CNTN1* significantly repressed the expression of cyclin D1 (CCND1), a target gene of the Notch1 pathway, indicating CNTN1 to potentially regulate the expression of CCND1 by activating Notch1 in thyroid cancer (19). Additionally, CNTN1 expression was significantly increased in RET/PTC3 transgenic mice and suppressed by the RET inhibitor regorafenib. Based on the profile database analysis, CNTN1 was identified as the new downstream molecule to the *RET/PTC3* fusion gene (19).

## Role of CNTN1 in Other Cancers

Next, the expression of CNTN1 was found to be positively associated with glioma and liver cancer, negatively with colorectal cancer and melanoma, and not associated with paraneoplastic neuropathies. The *CNTN1* was suggested to be a novel oncogene in glioblastoma (36). Its expression was associated with pilocytic astrocytoma located in the cerebellum (37), and it was defined as a new immunogenic tumor-associated antigen in isocitrate dehydrogenase (IDH)-mutant gliomas (38). Further, an elevated expression of CNTN1 was found in HCC and was associated with cancer progression and poor prognosis (20). Moreover, *CNTN1* was hypermethylated, and its expression was downregulated in colorectal cancer (39) and primary melanomas (40). Additionally, Siles et al. could not detect CNTN1 in immunoprecipitation experiments performed in sera from 34 patients with possible or definite paraneoplastic neuropathies (41). This may be explained by the relatively insufficient quantity of samples. Alternatively, CNTN1 was mainly associated with primary tumors with no effect on other conditions.

## Discussion

**Figure 1:**
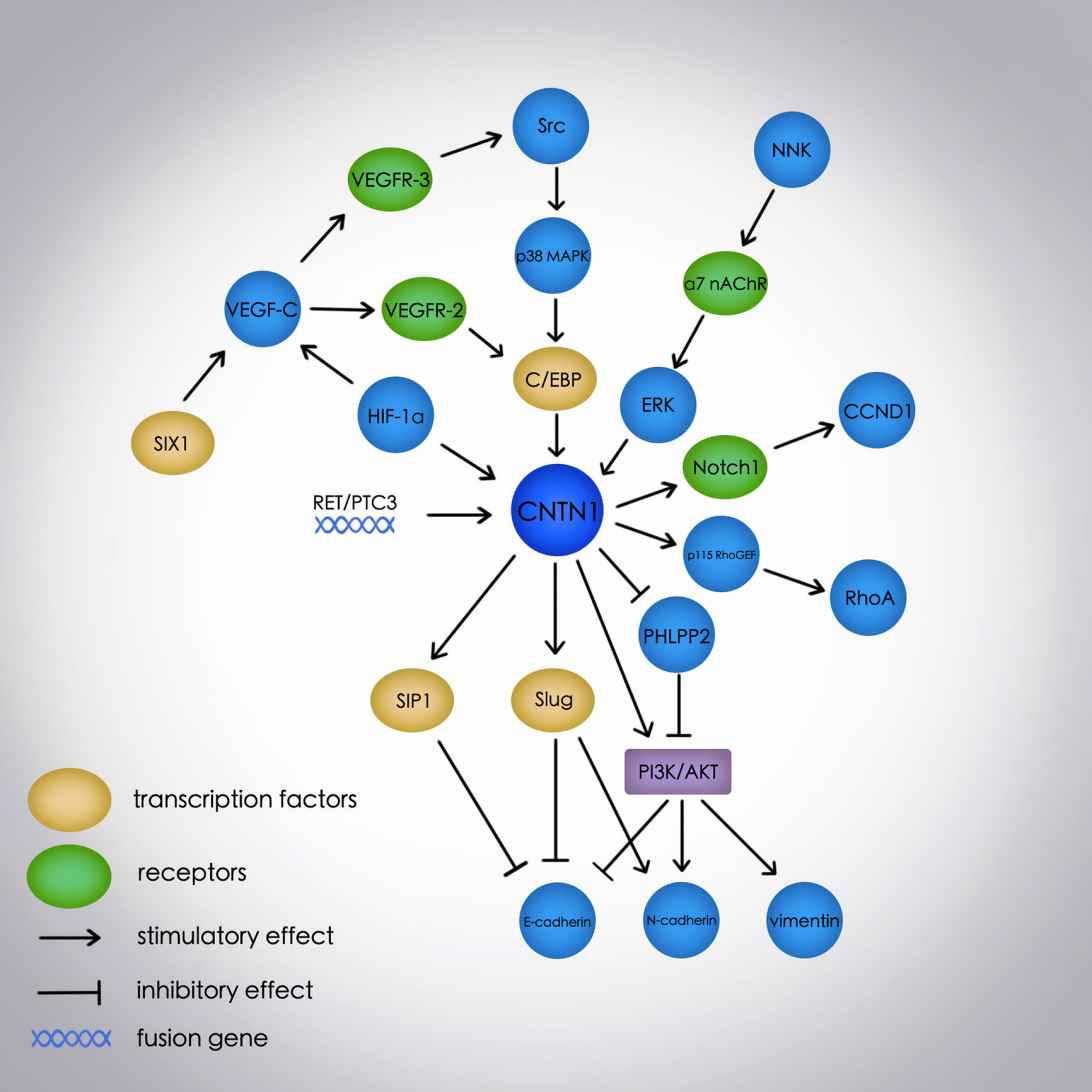
The molecular network of the EMT process and signal transduction pathways mediated by CNTN1 in cancers. E-cadherin, N-cadherin, and vimentin are EMT-related markers; E-cadherin is an epithelial marker, and N-cadherin and vimentin are mesenchymal markers. EMT, epithelial-mesenchymal transition; CNTN1, contactin 1; PI3K, phosphatidylinositol 3-kinase; PHLPP2, PH domain and leucine-rich repeat protein phosphatase 2; NNK, 4-(methylnitrosamino)-1-(3-pyridyl)-1-butanone; α7 nAChR, α7 nicotinic acetylcholine receptor; ERK, extracellular signal-regulated kinase; VEGF-C, vascular endothelial growth factor-C; VEGFR-3, vascular endothelial growth factor receptor-3; p38 MAPK, p38 mitogen-activated protein kinase; C/EBP, CCAAT/enhancer-binding protein; HIF-1α, hypoxia-inducible factor-1α; p115 RhoGEF, p115 Rho guanine nucleotide exchange factor; RhoA, member A of the Ras homolog gene family; SIX1, sine oculis homeobox homolog 1; VEGFR-2, vascular endothelial growth factor receptor-2; RET/PTC3, Ret protooncogene and Ret-activating protein ELE1; CCND1, cyclin D1.

Previous studies have determined the role of CNTN1 in neurogenesis, Alzheimer’s disease (42), multiple sclerosis (43, 44), and chronic inflammatory demyelinating polyneuropathy (45, 46) as a neuronal cell adhesion molecule and a component of septate-like junctions between the terminal myelin loops and axolemma (47). However, recent studies associate CNTN1 with EMT and several signal transduction pathways in numerous cancers (**Figure 1**). CNTN1 increases tumor cell invasion, migration, and metastasis by promoting EMT *via* activation of the PI3K/AKT pathway, downregulation of E-cadherin, and upregulation of N-cadherin and vimentin in lung and prostate cancers (14–16, 23, 26). Further, CNTN1-induced EMT is mediated by the transcription factor Slug but not Snail, suggesting a specific mechanism involving CNTN1 in cancers. Apart from the PI3K/AKT pathway, CNTN1 is also associated with the RhoA pathway in gastric cancer; VEGF-C/VEGFR-3 pathway in lung, gastric, and esophageal cancers, and OSCC; α7 nAChR/ERK and Src-p38 MAPK-C/EBPα pathways in lung cancer; and Notch1 and RET/PTC3 pathways in thyroid cancer. This shows that CNTN1 is broadly involved in several regulatory networks for tumor progression. Additionally, the overexpression of *CNTN1* activated the Notch pathway and inhibited neurogenesis (1, 3) while it regulated the expression of a Notch1 target gene. This shows the association between CNTN1 and Notch1 under different conditions. Furthermore, the transcription factor C/EBP is found to mediate the activity of CNTN1 associated with the VEGF-C/VEGFR-3 pathway in lung and esophageal cancers. This is a new aspect in tumor-related pathways although the exact mechanism remains to be studied in different cancers and pathways. Moreover, hypoxia-induced HIF-1α increases the expression of CNTN1, suggesting a probable link between VEGF-C/VEGFR-3 and RhoA pathways, mediated by HIF-1α and CNTN1. It is also reported that *CNTN1* knockdown influences genes involved in tumorigenesis although its overexpression affects genes associated with cancer, immunologic disorders, and inflammation (23).

In different cancer types, CNTN1 is positively associated with lymphatic invasion, metastasis, late TNM stage, and a short overall survival time although its role in proliferation remains controversial. The limitations of the study include a small sample size and limited cancer cell lines. However, the regulation of tumor proliferation is complicated with several molecules and signal transduction pathways. Therefore, in-depth investigations of molecular mechanisms are required to ascertain the role of CNTN1 in cancer cell proliferation.

Understanding the role of CNTN1 in lung cancer ranges from tumor progression to treatment. The expression of CNTN1 is correlated with drug resistance, and depletion of *CNTN1* reverses the drug sensitivity in lung cancer cell lines (13–15). These studies indicate CNTN1 as a potential therapeutic target, and the development of inhibitors and targeted drugs against CNTN1 may provide a new aspect in cancer research. Additionally, lung adenocarcinoma cells overexpressing *CNTN1* display well-formed F-actin-containing microfilament bundles, which could be abolished by knockdown of *CNTN1* (9). This indicates its critical role in regulating the rearrangement of F-actin-containing microfilament bundles and cancer invasion. Of note, overexpression of interleukin (IL)-8 and *CNTN1* is correlated with cachexia in non-small cell lung cancer, indicating a potential link between CNTN1 and immune response in cancers (48). Further, CNTN1 is identified as a novel immunogenic tumor-associated antigen in IDH-mutant gliomas (38). CNTN1 triggers the activation of frequent endogenous T-cell immune responses and is a potential T-cell target for immunotherapy. Additionally, IgG4 antibodies produced by regulatory B-cells disrupt the function of CNTN1 at the paranodal axoglial junctions without the involvement of inflammatory cells or complement. This suggests that antibody-mediated immunotherapy toward CNTN1 may be a novel direction in cancer treatment (49).

Thus, based on recent studies, CNTN1 is identified as a predictive biomarker and therapeutic target in several cancer types. However, clinical trials or targeted therapeutic drugs with regard to CNTN1 have not been established. Hence, the association between CNTN1 and other molecules and pathways warrants further investigations using an additional number of cell lines and *in vivo* studies. Moreover, targeted therapeutic drugs, immunotherapy, and clinical trials remain to be further studied.

## Author Contributions

YL designed and wrote the manuscript, CM and FL edited the table and the figure, GN reviewed the literature, and HZ edited the manuscript. All authors contributed to the article and approved the submitted version.

## Conflict of Interest

The authors declare that the research was conducted in the absence of any commercial or financial relationships that could be construed as a potential conflict of interest.

## Abbreviations

CNTN1, contactin 1; OSCC, oral squamous cell carcinoma; EMT, epithelial-mesenchymal transition; PI3K, phosphatidylinositol 3-kinase; VEGF-C, vascular endothelial growth factor-C; VEGFR-3, vascular endothelial growth factor receptor-3; nAChR, nicotinic acetylcholine receptor; ERK, extracellular signal-regulated kinase; RhoA, member A of the Ras homolog gene family; MAPK, mitogen-activated protein kinase; C/EBP, CCAAT/enhancer-binding protein; RET/PTC3, Ret protooncogene and Ret-activating protein ELE1; HCC, hepatocellular carcinoma; SCLC, small cell lung cancer; PHLPP2, PH domain and leucine-rich repeat protein phosphatase 2; PTEN, phosphatase and tensin homolog; HIF-1α, hypoxia-inducible factor-1α; SIX1, sine oculis homeobox homolog 1; NNK, 4-(methylnitrosamino)-1-(3-pyridyl)-1-butanone; RhoGEF, Rho guanine nucleotide exchange factor; CCND1, cyclin D1; IDH, isocitrate dehydrogenase; IL, interleukin; NSCLC, non-small cell lung cancer.
